# The Nutritional Value of Non-Traditional Gluten-Free Flakes and Their Antioxidant Activity

**DOI:** 10.3390/antiox8110565

**Published:** 2019-11-16

**Authors:** Kristýna Šťastná, Martina Mrázková, Daniela Sumczynski, Betül Cındık, Erkan Yalçın

**Affiliations:** 1 Department of Food Analysis and Chemistry, Tomas Bata University in Zlín, Vavrečkova 275, 760 01 Zlín, Czech Republic; kstastna@utb.cz (K.Š.); mmrazkova@utb.cz (M.M.); 2 Department of Food Engineering, Bolu Abant Ízzet Baysal University, Gölköy Campus, 14030 Bolu, Turkey; betul1506@hotmail.com (B.C.); erkanyalcin93@hotmail.com (E.Y.)

**Keywords:** antioxidant, ABTS, DPPH, *Oryza sativa* L., *Chenopodium quinoa* Willd, *Eragrostis tef* L., flake, HPLC, in vitro digestibility

## Abstract

Nowadays, there is a growing interest for foods with a lower sugar content and rich in fiber and biologically active substances. The main purpose of this study was to prepare flakes from non-traditional pigmented cereals (*Oryza sativa*, *Chenopodium quinoa,* and *Eragrostis* *tef*) and to analyze their fibre, sugar, and in vitro digestibility values. Regarding phenolic antioxidants (soluble, soluble conjugated, and insoluble bound fractions), their content and antioxidant activity were measured using spectrophotometry and high performance liquid chromatography (HPLC) methods. Hydrothermally treated grains resulted in flakes with higher total dietary fibre contents (11.1–24.4%), quinoa and teff flakes were rich in maltose (up to 42.0 mg/g). Non-traditional flakes had lower in vitro digestibility, but conversely, they exhibited the highest phenolic contents corresponding with the highest antioxidant activity values (up to 2.33 mg Gallic acid equivalent/g of total phenolic content and 1.59 mg Trolox equivalent/g for 2,2-diphenyl-1-picrylhydrazyl (DPPH) in case of brown teff). Among free phenolics, the main contributors to an antioxidant activity were *p*-coumaric, *o*-coumaric, and gallic acids (*r* > 0.8186); among the soluble conjugated fractions, they were epigallocatechin, epicatechin, caffeic, and vanillic acids (*r* > 0.5935); while caffeic, protocatechuic, and ferulic acids (*r* > 0.5751) were the main contributors among the insoluble bound phenolics.

## 1. Introduction

Rice (*Oryza sativa* L.), quinoa (*Chenopodium quinoa* Willd.), and teff (*Eragrostis tef*) belong to a group of gluten-free cereals or pseudocereals. White rice, quinoa, and teff grains are already widely used in diet around the world as a side dish or as a raw material for milling products which are then processed into bakery products and breakfast cereals or flakes [1,2]. Since the society is looking for new sources of nutrients, especially ones with high antioxidant activity, we can see a lot of new products on the market. These may also be flakes of wholegrain pigmented cereals and pseudocereals which are not widely used in European Union (EU), but it is known that they contain a high amount of minerals, vitamins, fibre, and other biological active compounds [3,4]. 

Epidemiological studies have shown that a diet rich in whole grains with low sugar amount and high content of phenolics, exhibiting antioxidant activity, is able to decrease the risk of diverse chronic diseases, such as cardiovascular and neurodegenerative, type II diabetes, or some types of cancer. An indisputable health advantage of these grains is the lack of gluten, which can cause celiac disease and gluten sensitivity [5,6,7]. Moreover, celiac disease is an immune-mediated disease characterized by a malabsorption of nutrients in gastrointestinal tract. As mentioned above, commonly consumed cereals and pseudocereals with higher fibre content can suit gluten-free diet requirements which is usually poor in alimentary nutrients such as polyphenol antioxidants [8]. These are important health consequences to cause developing healthy and value-added pigmented gluten free flakes with higher value of antioxidant activity. 

Only a few studies related to the preparation of gluten free flakes from grains with pigmented layers are available in the literature. Regarding the flaking process, thermal treatment affects the endosperm structure. Hydrothermal treatment is used to stabilize the products by inactivation of lipase and peroxidase enzymes. In addition, steaming modifies the hydration of macromolecular components of flakes, such as starch and proteins [9]. Although pigmented grains contain higher quantities of phenolics [10], it has already been confirmed that their amount in whole grain decreases during the hydrothermal treatment [11,12]. Studies of cereals usually aim only at soluble fraction of phenolics which are absorbed in the digestive system right into the blood stream. However, insoluble bound phenolics are also important in consumer’s health. As they are bound to insoluble macromolecules of food matrix, they have to be released firstly by fermentation by microorganisms of large intestine. Once released they influence the colon environment which leads to better protection against harmful bacteria [13]. Nevertheless, the detailed research is needed for involving the nutritional composition and antioxidant activity of non-traditional gluten-free flakes.

Following the above information about health effects and unsatisfactory research on non-pigmented gluten-free cereal flakes, the aim of this study was firstly targeted on preparation of cereal flakes using hydrothermal treatment from non-traditional cereals or pseudocereals (white, red, and black rice; white, red, and black quinoa; white and brown teff). To assess the potential health effect of the product, determination of essential nutritional composition, and in vitro digestibility was performed. Furthermore, free, soluble conjugated and insoluble bound phenolics were extracted to measure total flavonoid and polyphenolic contents, antioxidant activity and individual profile using spectrophotometric and high performance liquid chromatography (HPLC) methods, respectively. Then, appropriate correlations between nutrients and digestibility values and also between phenolic compounds and antioxidant activity were evaluated.

## 2. Materials and Methods 

### 2.1. Preparation of Non-Traditional Gluten-Free Flakes

Grain samples of white, red, and black varieties of *Oryza sativa* L. (originated from China, Cambodia and Italy, respectively) and *Chenopodium quinoa* Willd. (all originated from Peru), and white and brown varieties of *Eragrostis tef* L. (originated from Bolivia and EU, respectively) were bought from local markets in Zlín (Czech Republic) in the amount of five packages of 200–400 g from each sample. All commercial grain samples, therefore, without knowing their variety, were purchased in the period of 2017–2018. Latin names and countries of origin of grains have been taken from packages. 

The sample preparation stages were as follows: 5 × 0.15 kg of each sample were directly cooked in unsalted water adjusted to temperature of 95–96 °C for 15–19 min for rice grains, for 8–9 min for quinoa grains, and for 17 min for teff. The cooking time corresponded to completion of starch gelatinization. After cooking, water was removed and cooked grains were conditioned in a covered beaker (rice grains for 45 min, quinoa and teff for 25 min). Afterward, all grain samples were air-dried at room temperature for 100–120 min; and then rice flakes were prepared using a Combi-Star grinder (Waldner Biotech, Lienz, Austria) equipped with a flake roller and quinoa and teff samples were rolled manually by a wooden roller. All flake samples were dried out in a laboratory oven at 30 °C for 120 min until dry matter content reaches up to 85% [14]. Prepared flakes differed in shape and size. Rice flakes had an oval shape, and the sizes of red and black rices were 6 × 2 and 5 × 3 mm, respectively. The shape of flakes prepared from quinoa and brown teff were much more circular with diameters of 3 and 1 mm, respectively. Samples were stored in non-transparent plastic bottles at ambient temperature for only two weeks. Each sample was milled using a grinder (Combi-Star, Waldner Biotech, Lienz, Austria) before analysis.

### 2.2. Chemicals and Reagents

Trifluoracetic acid, acetonitrile, methanol, and ethanol were provided from Penta (Prague, Czech Republic). α-Amylase and neutral detergent solution package (containing sodium lauryl sulphate, EDTA disodium, sodium borate, sodium phosphate dibasic together with triethylene glycol) were purchased from Ankom Technology (Macedon, NY, USA). Folin-Ciocalteu reagent, AlCl_3_·6H_2_O were purchased from Penta (Prague, Czech Republic). The substance 2,2’-azinobis(3-ethylbenzo-thiazoline-6-sulfonic acid) diammonium salt (ABTS), radical 2,2-diphenyl-1-picrylhydrazyl (DPPH), and standard 6-hydroxy-2,5,7,8-tetramethylchroman-2-carboxylic acid (trolox) were provided from Sigma-Aldrich (St. Louis, MI, USA). Phenolic and sugar standards were as follows: Epigallocatechin, catechin, epicatechin, rutin, quercetin, kaempferol, neochlorogenic, chlorogenic, gallic, protocatechuic, *p*‑hydroxybenzoic, vanillic, caffeic, syringic, *p*-coumaric, ferulic, sinapic, ellagic, *o*-coumaric, cinnamic acids and protocatechuic acid ethyl ester; D(+)-maltose, D(+)-glucose, D(−)-fructose, D(+)-rhamnose, D(+)-xylose, D(+)-saccharose, all were purchased from Sigma-Aldrich (St. Louis, MI, USA). All standards and solvents used in this study were of HPLC-grade (purity ≥ 98.5–99.0%). Total dietary fiber and resistant starch assay kits were provided from Megazyme International Ireland Ltd. (Wicklow, Ireland). 

### 2.3. Chemical Composition Analysis 

Determination of ash and dry matter contents were carried out using the methods of Association of official analytical chemists (AOAC) [14,15]. 

For the determination of free sugars the milled samples (5 g of each) were extracted with 40 mL of redistilled water (30 °C) by stirring on the magnetic stirrer for 30 min. Then, the volumetric flask (50 mL) was filled up to mark with redistilled water and the sample mixture was filtered through KA 4 of filter paper [16]. Individual sugars were determined using the HPLC system (Dionex Ultimate 3000, Thermo Scientific; Waltham, MA, USA) coupled to a detector (ERC RefractoMax 520, Thermo Scientific; Waltham, MA, USA). Data signals were analyzed by LC Chromeleon^TM^ 7.2 software (Thermo Scientific, Waltham, MA, USA). The chromatographic separation was carried out using a Rezex RCM-Monosaccharide Ca^2+^ column (100 × 7.8 mm; 8 μm, Phenomenex, Torrance, CA, USA). Redistilled water was used as a mobile phase in isocratic elution mode for 15 min. The injection volume was 15 µL, flow rate 0.4 mL/min, and temperature of column was set at 80 °C. Chromatogram was recorded with linear responses within the calibration range of 0.5–10.0 mg/mL with correlation coefficients exceeding 0.9996. Individual sugars were identified according to the retention time of standards. 

Crude fibre (CF) and neutral-detergent fibre (NDF) contents, and in vitro organic matter (OMD) and dry matter (DMD) digestibility values were measured according to Sumczynski, Bubelová, and Fišera (2015) with a two-step enzymatic procedure using pepsin and pancreatin [17]. Total dietary fibre (TDF) contents of milled flake samples were determined according to Megazyme Total Dietary Fiber Assay Procedure which is based on the methods of American Association of Cereal Chemist International (AACCI) Approved Methods of Analysis Methods No. 32-05.01, 32-06.01, 32-07, and 32-21.01 [18]. Resistant starch (RS) contents of milled flake samples were determined according to the Megazyme Resistant Starch Assay Procedure which is based on the methods of AACCI Approved Methods of Analysis Method No. 32-40.01, and AOAC Method 2002.02 [18,19]. 

### 2.4. Extraction of Phenolic Antioxidants

#### 2.4.1. Extraction of Free Phenolics

Free phenolics were extracted using the method reported by Qiu, Liu, and Beta (2010) with some modifications [20]. The milled sample was weighed as 1.5 g into four polypropylene tubes and then 15 mL of 80% methanol was added to each tube. The mixture was extracted on a mechanical shaker for 1 h at room temperature. The extracts were kept in separate glass flasks. The extraction was repeated by adding 10 mL of 80% methanol and then extracts were combined. The collected extracts were concentrated to dryness by using laboratory rotary evaporator. Two of four dried methanol extracts were re-dissolved by using 10 mL of 50% methanol, then were quantitatively poured into a 10 mL volumetric flask and a possible precipitate was dissolved by using an ultrasonic bath. 

#### 2.4.2. Extraction of Soluble Conjugated Phenolics

The two remaining dried methanol extracts were dissolved by using 10 mL of 4 M NaOH and quantitatively poured into a vial. This solution was hydrolysed under nitrogen gas on a mechanical shaker for 4 h at room temperature. The pH of the extract of conjugated phenolics was then set to pH between 3 and 5 by using 6 M HCI. 

#### 2.4.3. Extraction of Insoluble Bound Phenolics

To extract insoluble bound phenolics, 20 mL of 4 M NaOH was added onto a solid residue in remaining two vials. The mixture was hydrolysed under nitrogen gas on a mechanical shaker for 4 h at room temperature. After hydrolysis, the pH of the mixture was set to pH between 3 and 5 by using 6 M HCl and then the mixture was centrifuged (Velocity 13 μ; Dynamica Scientific Ltd., Newport Pargnell, UK) at 12,300× *g* for 15 min. 

### 2.5. Determination of Total Flavonoid, Total Phenolic Contents, and Antioxidant Activity

All fractions (free, conjugated, and bound) were directly used in total flavonoid and polyphenol contents and antioxidant activity assays. The total flavonoid contents (TFC) and total phenolic contents (TPC) were determined in the spectrophotometer using AlCl_3_ and Folin-Ciocalteu reagents [21]. In case of TFC, rutin was used as a standard (0 to 1.4 mg/mL); gallic acid (0 to 1000 mg/L) was used as a reference standard for determination of TPC. The antioxidant activities were determined according to Sumczynski et al. (2015) using ABTS and DPPH radicals where trolox (0 to 300 mg/L) as a reference standard was used [21].

### 2.6. Determination of Individual Phenolics using HPLC

The content of phenolic compounds was determined using an HPLC system (Thermo Scientific Dionex Ultimate 3000, Waltham, MA, USA) equipped by diode array detector (DAD-3000 RS, Thermo Scientific Dionex UltiMate 3000, Waltham, MA, USA). The phenolic content was detected according to Kotásková, Sumczynski, Mlček, and Valášek (2016) with some modifications [22]. Phenolic acids were separated using a Kinetex column C18 (150 × 4.5 mm; 2.6 mm, Phenomenex; Torrance, CA, USA). In the analysis, 10 μL of sample was injected into the column. For the gradient elution following mobile phases were used—(A) redistilled water:acetic acid in the ratio of 99:1 and (B) redistilled water:ACN:acetic acid in the ratio of 67:32:1. The gradient was programmed as follows: 10% B at 0 min; 20% B between 0 and 10 min; 20–40% B between 10 and 16 min; 40–50% B between 16 and 25 min; 50–70% B between 25–26 min; 70% B between 26–30 min; 70–10% B between 30 and 40 min, and 10% B between 40 and 45 min. The other parameters included: The flow rate to 1 mL/min, the column temperature to 30 °C, and the wavelength 275 nm for recording the chromatograms. In the calibration range of 5.0–50.0 μg/mL the DAD response was linear for all phenolics. Correlation coefficients for all selected phenolics exceeded 0.9995. The individual phenolic compound was identified according to the retention time obtained from the chromatogram of corresponding standard. 

### 2.7. Statistical Analysis

All analyses were repeated five times and the results are reported as mean ± standard deviation (SD) on dry weight basis. The results were statistically evaluated using one-way analysis of variance (ANOVA). Tukey´s test was subsequently applied to identify differences among means. The level of confidence was set to 95% (*p* < 0.05). Correlations between the data were defined using Pearson´s correlation coefficients (*r*). 

## 3. Results and Discussions

### 3.1. Chemical Composition

The contents of dry matter, ash, individual sugars, CF, NDF, and TDF and values of OMD and DMD are presented in Table 1. The dry matter content ranged between 90.0% and 93.3%. The determination of this parameter is important in managing and marketing flakes, and also in correcting flake composition on dry weight basis. The ash contents of non-traditional flakes ranged from 1.00% to 2.10%. It could be assumed that quinoa and teff flakes might serve as a good source of minerals.

As can be seen in Table 1, the predominant sugar in all flake samples was maltose with the concentration ranging from 4.91 to 42.0 mg/g. The highest maltose content was detected in red quinoa flakes (42.0 mg/g) followed by black quinoa flakes (39.4 mg/g). Regarding glucose and fructose amounts, their concentration ranged between 0.09 to 4.60 and 0.08 to 1.38 mg/g, respectively. Red and black quinoa flakes were not only rich in maltose, but also in glucose and fructose contents. Moreover, rhamnose was detected only in white rice flakes (below 0.02 mg/g) and xylose was found only in red rice flakes (0.03 mg/g). Saccharose was not found in any sample. A typical HPLC chromatogram can be seen in Supplementary Figure S1. As far as our knowledge, no studies have been stated on the free sugar profile of hydrothermally treated non-tradition flakes with coloured layers. Nevertheless, Hu et al. (2017) provided an approximate maltose content in milled rice grains up to 1.34 mg/g, fructose content up to 0.11 mg/g, and glucose content up to 2.29 mg/g [16]. Concentrations of sucrose, glucose, and fructose in quinoa seeds were the following: Up to 15.2, 8.0, and 1.6 mg/g, respectively [23]. It might be assumed that the high maltose content in non-traditional flakes could point to degradation of starch during hydrothermal treatment of grains. 

The crude fibre (CF, complex of lignin, and cellulose) and neutral-detergent fibre (NDF, complex of lignin, cellulose, and insoluble part of hemicelluloses) contents ranged from 1.27% to 1.93% and 2.39% to 8.95%, respectively. Total dietary fibre (TDF, complex of insoluble and soluble parts of fibre) was measured in the range of 7.30–24.4%, with the highest TDF content in red quinoa flakes. It has been shown that hydrothermal treatment of raw grains increased digestibility of both OMD and DMD value as well as effectively improved the nutritional value of grains by pioneering starch gelatinization. According to Qiao et al. (2015) the OMD values of intact rice grains increased by 5% (from 68.8% to 73.4%) when steam-flaked process was applied [24]. In this study, the OMD values of non-traditional flakes ranged from 89.2% to 98.4%. It was found that rice flakes were more digestible than quinoa and teff flakes. When the CF values were high, DMD and OMD values exhibited as low, it could be considered that they were positively correlated as *r* (Pearson´s correlation coefficient) values of 0.3349 and 0.4786, respectively (Supplementary Table S1). In case of NDF and TDF values, it could not be seen any positive correlations with DMD or OMD values. Moreover, the positive correlations were also observed between high starch or resistant starch contents and DMD or OMD values, in which *r* values were between 0.2911–0.7755.

### 3.2. Results of Total Phenolic and Flavonoid Contents and Antioxidant Activity Values

Results of total flavonoids and phenolics in free, soluble conjugated and insoluble bound fractions of non-traditional flakes are shown in Table 2. The highest TFC content was observed in red quinoa flakes (1.06 mg RE/g), where flavonoids mostly concentrated in the free fraction (0.57 mg RE/g). Same situation was also seen in white and black quinoa flakes. The highest TFC content in soluble conjugated fraction was measured in white teff flakes (0.42 mg RE/g), in case of insoluble bound fraction, the highest TFC content was detected in brown teff flakes (0.32 mg RE/g). It may also be pointed out that the highest TFC content was found in insoluble bound fraction of red rice flakes, that value was 73% of total TFC. 

Focusing on TPC values, the highest TPC values were observed in brown teff flakes (2.33 mg GAE/g) followed by red quinoa flakes (2.32 mg GAE/g). It could be concluded that insoluble bound fraction of all rice flakes was rich in TPC content, as in the range of 0.27–0.59 mg GAE/g, whereas free fractions of white and brown teff flakes were rich in TPC as 0.91 and 1.01 mg GAE/g, respectively. In case of quinoa samples, flakes with colour layers were rich in free TPC concentrations (0.87 and 0.85 mg GAE/g), while in case of white type quinoa flakes, the highest part of TPC was measured in the insoluble fraction (specifically 38% of total TPC). The concentrations of phenolics are naturally significant in pigmented types of grains [3,10,23]. It must also be considered that currently available composition data on some pigmented grains and flakes are difficult to compare since researches used different extractions and each one was modified for one or more grain varieties. Moreover, the reactivity of Folin-Ciocaulteu reagent with other non-phenolic substances has to be taken into the consideration [23]. In addition, not much research has been conducted to evaluate the contents of TPC and TFC values in all three fractions of non-traditional flakes. 

In this study, two antioxidant assay procedures were applied using ABTS and DPPH free radicals in order to express the antioxidant property with two different mechanisms. The results of antioxidant properties are shown in Table 3. The TE values of non-traditional pigmented flakes obtained using ABTS and DPPH radicals varied from 0.66 to 1.24 and from 1.18 to 1.59 mg TE/g, respectively. Non-pigmented flakes were poor in antioxidant properties compared to pigmented varieties. Similar results were also observed in some studies of antioxidant activity of pigmented and non-pigmented grains [11,23,25]. It is clear that thermal processes lead to decomposition of phenolics and decrease in antioxidant activity values, for instance this fact was demonstrated in application of parboiling process on pigmented rice grains [3,12]. The results of ABTS and DPPH scavenging activity found in individual phenolic fractions were consistent mainly with TPC results (Supplementary Table S1). The results showed a positive linear correlation between antioxidant activity assays and TPC contents in all fractions while applying ABTS (*r* = 0.6487–0.9861) and DPPH (*r* = 0.3795–0.9023) radicals. Conversely, non-linear correlation between TFC contents and antioxidant activities was confirmed for soluble conjugated fraction of flavonoids (*r* = –0.0695) when DPPH free radical was applied.

### 3.3. Free, Soluble Conjugated and Insoluble Bound Phenolic Compounds Detected using HPLC

Determining the contents of free, soluble conjugated and insoluble bound phenolic fractions is actually very important from the nutritional point of view. Especially, insoluble bound phenolic fraction arrives to the colon as an intact form. After the fermentation of polysaccharides of cell wall to which they are bound to, they become accessible to colonic microflora and intestinal enzymes [3]. Major individual phenolics in each fraction are presented in Table 4, Table 5 and Table 6, and total individual phenolic concentrations are also exhibited in Supplementary Table S2. Chromatograms of individual phenolics for black quinoa flakes can be seen in Supplementary Figures S2–S4. 

Regarding free phenolics, high contents of epigallocatechin (46.90 µg/g), rutin (129.0 µg/g), and sinapic acid (47.20 µg/g) were found in red quinoa flakes, while ferulic acid (150 µg/g) was the most abundant instead of sinapic acid in black quinoa flakes (Table 4). Concerning the teff flakes, white teff was rich in epigallocatechin (45.50 µg/g) compared with brown variety where rutin (43.50 µg/g) and o-coumaric acid (49.70 µg/g) were predominant phenolics in free fractions. It is worth to notice that high contents of protocatechuic acid in black and red rice flakes (36.80 and 26.20 µg/g, respectively) were recorded. Moreover, quercetin was not detected in white and black rice flakes as well as in teff flakes, neochlorogenic acid was also not assessed in red and black rice flakes, similar to white quinoa flakes. Gallic, *p*-hydroxybenzoic, ellagic, *p*-coumaric, and cinnamic acids were not detected in red quinoa flakes. 

The soluble conjugated phenolic contents of non-traditional flakes are shown in Table 5. High concentrations of epigallocatechin were detected in flakes prepared from quinoa and teff grains (changed between 31.60 and 261.0 µg/g). In addition, white teff flakes significantly had the highest rutin content (81.0 µg/g). In case of soluble conjugated phenolic acids, high amounts of protocatechuic, vanillic, and ferulic acids were detected in quinoa flakes, while neochlorogenic and syringic acids dominated in teff flakes. The high concentration of sinapic acid (43.20 µg/g) was observed in soluble conjugated phenolic fraction of black rice flakes. Cinnamic acid was not detected in all rice and teff and red quinoa flake samples. Ferulic and *p*-coumaric acids were not found in red and black rice flakes and ellagic, *o*-coumaric acids and protocatechin ethyl ester were not detected only in red rice flakes. Similarly, kaempferol was not recorded in rice and teff flakes, quercetin was not measured in all rice and brown teff flakes. 

Insoluble bound phenolics are presented in Table 6. Ferulic acid, which was detected in red and black rice flakes (228.0 and 257.0 µg/g, respectively), was the major insoluble bound phenolic acid as well as in teff samples. When the insoluble bound flavonoids were considered, high concentrations of epigallocatechin were detected in red and black quinoa and brown teff flakes (35.90, 40.20, and 45.50 µg/g, respectively). In addition, brown and white teff flakes were rich in catechin (31.80 and 62.30 µg/g, respectively) as well as in quercetin (28.60 and 43.60 µg/g, respectively). Similarly, high contents of quercetin were determined in red and black rice flakes. White and brown teff flakes further contained high amounts of sinapic acid (26.2 and 47.5 µg/g, respectively) and red rice flakes had the highest content of ellagic acid (15.40 µg/g). Insoluble bound cinnamic acid was detected only in black quinoa and brown teff flakes. 

Literature data related to phenolics of flake products produced from non-traditional cereals and pseudocereals are not very frequent. It must also be highlighted that currently available composition data on some pigmented grains do not give concrete information, since researchers used different extraction methods for obtaining bioactive substances. Therefore, the polyphenol compound composition is much dependent on grain variety. Based on the above consideration, some results from thermally treated grain measurements were postulated. It was reported in many studies that heat treatment in the presence of moisture (e.g., steaming, boiling, parboiling) resulted in the reduction of the phenolic concentrations [3,12,26]. The main reason responsible for decreasing in phenolic contents after hydrothermal treatment was reported as changing of chemical structure of grain [12]. Additionally, other factors have an effect on the presence of phenolic compounds, e.g., environmental and agrotechnical conditions or the heat treatment processes. Scaglioni et al. (2014) detected chlorogenic and *p*-coumaric acids as the main phenolic acids in free fractions of parboiled rice grains, while insoluble bound phenolic fraction of same grains included the high amounts of ferulic acid [12]. The major free phenolics determined in hydrothermally treated white teff grains were epigallocatechin, rutin, ellagic, *p*-coumaric and gallic acids, while epigallocatechin, rutin, gallic, protocatechuic, *p*-coumaric and ferulic acids were the lead phenolics in brown teff grains. Concerning insoluble bound phenolics, white teff contained high values of catechin, epigallocatechin, ferulic, sinapic and ellagic acids, whereas catechin, gallic, vanillic, ferulic and sinapic acids were the main phenolics in brown teff grains [26]. The principal polyphenolic acids of quinoa grains were reported as caffeic, ferulic, *p*-coumaric, gallic and protocatechuic acids [27].

To discover the main contributors to the antioxidant activity in each fraction the correlations between individual phenolic values and antioxidant activities were calculated. Corresponding correlation coefficients are displayed in Supplementary Table S3. In terms of individual phenolics in free fractions, the main contributors to antioxidant activity seemed to be *p*-coumaric acid > *o*-coumaric acid > gallic acid > vanillic acid > ellagic acid > epigallocatechin > syringic and sinapic acids with the correlation coefficients *r* ranging from 0.9493–0.3322. Regarding the soluble conjugated phenolic fractions, the main contributors to antioxidant activity were epigallocatechin > caffeic acid > vanillic acid > protocatechuic acid > epicatechin > rutin and gallic acid (*r* in the range of 0.6906–0.3677, respectively). Considering the insoluble bound phenolic fractions, the main contributors to antioxidant activities were caffeic acid > protocatechuic acid > epigallocatechin > quercetin > epicatechin > sinapic acid > gallic and ferulic acids with the correlation coefficients between 0.8253 and 0.3759, respectively. 

It is widely known that different kinds of interaction between phenolics and free radicals depend on their chemical structure, on specific antioxidant interactions in the tested system such as extraction procedures, etc. Moreover, there are also synergistic and antagonistic interactions between phenolic compounds and other antioxidants or substances included in the matrix of flakes. Clearly, theoretical correlations cannot be accurately reflected in the metabolic pathways in the gastrointestinal tract. In reality other factors as consumption of glycoside forms of phenolics, enzyme activity or digestive factors have influence on these interactions [28,29,30].

## 4. Conclusions

To prepare and determine nutritional properties of non-traditional gluten-free flakes, pigmented types of rice, quinoa, and teff grains were subjected to hydrothermal treatment process. This study confirmed that non-traditional pigmented grain samples can be used to produce flakes with higher ash, dietary fibre, and free sugar contents. Especially, high concentrations of maltose were measured in quinoa and teff flakes. Pigmented flakes exhibited higher TPC and TFC values and in vitro antioxidant activity due to free, soluble conjugated and insoluble bound phenolic fractions. Regarding individual phenolic compound, the main flavonoids and phenolic acids in each phenolic fraction of pigmented flakes were measured. Correlations between antioxidant radical scavenging activity values and individual phenolics were evaluated to find out the main contributors to the antioxidant activity from the free, soluble conjugated and insoluble bound phenolic fractions. In the free phenolic fractions, *p*-coumaric, *o*-coumaric, gallic, vanillic and ellagic acids were the most important contributors to antioxidant activity of pigmented gluten-free flakes. In the soluble conjugated fraction, they were epigallocatechin, epicatechin, caffeic, vanillic and protocatechuic acids. Caffeic, protocatechuic, sinapic, gallic and ferulic acids, and epigallocatechin with quercetin were significant contributors to antioxidant activity in the insoluble bound fractions. When all individual phenolics were considered, epigallocatechin, catechin, rutin, sinapic and gallic acids seemed to be the first five major contributors to antioxidant activity in non-traditional pigmented flakes.

## Figures and Tables

**Table 1:** Results of basic nutrition parameters and free sugar contents in non-traditional flakes.

Values	White Rice	Red Rice	Black Rice	White Quinoa	Red Quinoa	Black Quinoa	White Teff	Brown Teff
	Nutrition parameters							
Dry matter (%)	90.0 ± 0.5 ^a,A^	90.5 ± 0.5 ^a,b,A,B^	90.9 ± 0.2 ^b,B^	91.0 ± 0.5 ^b,d,A^	93.3 ± 0.3 ^c,B^	92.8 ± 0.3 ^c,B^	91.7 ± 0.4 ^d,A^	90.4 ± 0.6 ^a,B^
Ash (%)	1.00 ± 0.05 ^a,A^	1.52 ± 0.02 ^b,B^	1.80 ± 0.10 ^c,C^	1.90 ± 0.05 ^c,A^	2.10 ± 0.04 ^d,e,B^	2.00 ± 0.04 ^e,B^	2.00 ± 0.10 ^e,A^	2.10 ± 0.10 ^e,A^
RS (%)	0.32 ± 0.03 ^a,A^	0.46 ± 0.01 ^b,B^	0.17 ± 0.01 ^c,C^	0.73 ± 0.06 ^d,A^	0.26 ± 0.02 ^e,B^	0.32 ± 0.02 ^a,C^	0.59 ± 0.06 ^f,A^	0.82 ± 0.01 ^g,B^
CF (%)	1.27 ± 0.04 ^a,A^	1.63 ± 0.04 ^b,B^	1.73 ± 0.03 ^c,d,C^	1.68 ± 0.04 ^b,d,A^	1.17 ± 0.03 ^e,B^	1.55 ± 0.05 ^f,C^	1.86 ± 0.10 ^g,A^	1.93 ± 0.05 ^g,A^
NDF (%)	2.39 ± 0.10 ^a,A^	3.12 ± 0.05 ^b,B^	3.25 ± 0.10 ^b,B^	7.21 ± 0.15 ^c,A^	7.73 ± 0.10 ^d,B^	8.95 ± 0.22 ^e,C^	6.55 ± 0.10 ^f,A^	6.93 ± 0.10 ^g,B^
TDF (%)	7.30 ± 0.10 ^a,A^	12.2 ± 0.3 ^b,B^	11.1 ± 0.4 ^c,C^	14.8 ± 0.3 ^d,A^	24.4 ± 0.3 ^e,B^	22.3 ± 0.4 ^f,C^	14.4 ± 0.3 ^d,A^	15.7 ± 0.2 ^f,B^
DMD (%)	97.4 ± 0.5 ^a,A^	94.9 ± 0.3 ^b,B^	95.2 ± 0.3 ^b,c,B^	95.9 ± 0.5 ^c,A^	87.1 ± 0.2 ^d,B^	87.3 ± 0.6 ^d,C^	94.2 ± 0.3 ^e,A^	93.5 ± 0.5 ^e,A^
OMD (%)	98.4 ± 0.3 ^a,A^	96.0 ± 0.5 ^b,e,B^	96.6 ± 0.4 ^b,B^	97.1 ± 1.0 ^c,A^	89.20 ± 0.4 ^d,B^	89.6 ± 0.5 ^d,B^	95.6 ± 0.2 ^e,A^	96.0 ± 1.0 ^e,A^
	Free sugars							
Glucose (mg/g)	0.16 ± 0.02 ^a,A^	0.09 ± 0.01 ^b,B^	2.14 ± 0.10 ^c,C^	1.36 ± 0.05 ^d,A^	4.60 ± 0.03 ^e,B^	3.91 ± 0.10 ^f,C^	2.05 ± 0.02 ^c,g,A^	1.98 ± 0.04 ^g,A^
Fructose (mg/g)	0.08 ± 0.01 ^a,A^	0.12 ± 0.01 ^b,B^	0.14 ± 0.03 ^c,C^	0.40 ± 0.05 ^d,A^	1.38 ± 0.03 ^e,B^	1.15 ± 0.02 ^c,C^	0.98 ± 0.04 ^f,A^	1.06 ± 0.02 ^g,B^
Rhamnose (mg/g)	≤0.02	ND	ND	ND	ND	ND	ND	ND
Xylose (mg/g)	ND	0.03 ± 0.01	ND	ND	ND	ND	ND	ND
Maltose (mg/g)	4.59 ± 0.10 ^a,A^	4.91 ± 0.12 ^b,B^	21.2 ± 0.2 ^c,C^	28.2 ± 0.2 ^d,A^	42.0 ± 0.2 ^e,B^	39.4 ± 0.1 ^f,C^	22.1 ± 0.2 ^g,A^	23.4 ± 0.3 ^h,B^
Saccharose (mg/g)	ND	ND	ND	ND	ND	ND	ND	ND

Results are presented in dry weight as means ± SD, *n* = 5. Means within the line with at least one identical small superscript (in case of all types of flakes) and large superscript (in case of each type group of flakes) do not differ significantly (*p* ≥ 0.05), while means with various superscripts show a significant difference (*p* < 0.05). RS—Resistant starch, CF—Crude fibre, NDF—Neutral-detergent fibre, TDF—Total dietary fibre, DMD—Dry matter digestibility, OMD—Organic matter digestibility. ND—Not detected: Value of LOD rhamnose and xylose—0.005 mg/mL, saccharose—0.002 mg/mL.

**Table 2:** Results of total flavonoid and phenolic contents in free, soluble conjugated and insoluble bound phenolic fractions of non-traditional flakes.

Phenolics	White Rice	Red Rice	Black Rice	White Quinoa	Red Quinoa	Black Quinoa	White Teff	Brown Teff
Free TFC	0.16 ± 0.01 ^a,A^	0.29 ± 0.01 ^b,B^	0.31 ± 0.01 ^c,C^	0.22 ± 0.03 ^d,A^	0.57 ± 0.02 ^e,B^	0.44 ± 0.03 ^f,C^	0.32 ± 0.03 ^c,A^	0.36 ± 0.02 ^h,B^
	33%	25%	54%	45%	54%	48%	32%	37%
Soluble conjugated TFC	0.14 ± 0.01 ^a,A^	0.05 ± 0.02 ^b,B^	0.06 ± 0.02 ^b,B^	0.12 ± 0.01 ^c,A^	0.27 ± 0.03 ^d,B^	0.28 ± 0.02 ^d,B^	0.42 ± 0.01 ^e,A^	0.30 ± 0.01 ^f,B^
	28%	2%	10%	24%	25%	30%	42%	31%
Insoluble bound TFC	0.19 ± 0.02 ^a,e,A^	0.25 ± 0.01 ^b,B^	0.21 ± 0.01 ^c,e,C^	0.15 ± 0.01 ^d,A^	0.22 ± 0.03 ^c,B^	0.20 ± 0.01 ^e,C^	0.27 ± 0.01 ^f,A^	0.32 ± 0.01 ^g,B^
	39%	73%	36%	31%	21%	22%	26%	32%
Total TFC	0.49 ± 0.02 ^a,A^	0.59 ± 0.02 ^b,B^	0.58 ± 0.02 ^b,B^	0.49 ± 0.03 ^a,A^	1.06 ± 0.03 ^c,B^	0.92 ± 0.02 ^d,C^	1.01 ± 0.02 ^e,A^	0.98 ± 0.02 ^f,B^
Free TPC	0.20 ± 0.07 ^a,A^	0.39 ± 0.01 ^b,B^	0.41 ± 0.04 ^c,C^	0.35 ± 0.01 ^d,A^	0.87 ± 0.05 ^e,B^	0.85 ± 0.04 ^e,B^	0.91 ± 0.08 ^f,A^	1.01 ± 0.04 ^g,B^
	31%	34%	35%	27%	38%	40%	52%	44%
Soluble conjugated TPC	0.18 ± 0.02 ^a,A^	0.16 ± 0.01 ^b,B^	0.23 ± 0.01 ^c,C^	0.45 ± 0.05 ^d,A^	0.64 ± 0.02 ^e,B^	0.65 ± 0.03 ^e,B^	0.53 ± 0.02 ^f,A^	0.57 ± 0.04 ^g,B^
	28%	14%	20%	35%	28%	30%	28%	24%
Insoluble bound TPC	0.27 ± 0.01 ^a,A^	0.59 ± 0.02 ^b,B^	0.53 ± 0.01 ^c,C^	0.48 ± 0.04 ^d,A^	0.81 ± 0.07 ^e,B^	0.63 ± 0.04 ^f,C^	0.31 ± 0.03 ^g,A^	0.75 ± 0.02 ^h,B^
	41%	52%	45%	38%	34%	30%	20%	32%
Total TPC	0.65 ± 0.03 ^a,A^	1.14 ± 0.02 ^b,B^	1.17 ± 0.03 ^c,C^	1.28 ± 0.04 ^d,A^	2.32 ± 0.06 ^e,B^	2.13 ± 0.04 ^f,C^	1.75 ± 0.04 ^g,A^	2.33 ± 0.04 ^e,B^

Results are presented in dry weight as means ± SD, *n* = 5. Means within the line with at least one identical small superscript (in case of all types of flakes) and large superscript (in case of each type group of flakes) do not differ significantly (*p* ≥ 0.05), while means with various superscripts show a significant difference (*p* < 0.05). The percentage contributions of free, soluble bound and insoluble bound fractions to the total flavonoid and phenolic contents are presented in brackets. TFC—Total flavonoid content (mg RE/g), TPC—Total phenolic content (mg GAE/g), RE—Rutin equivalent, GAE—Gallic acid equivalent.

**Table 3:** Results of antioxidant activity assay in free, soluble and insoluble bound phenolic fractions of non-traditional flakes.

Antioxidant Activity (mg TE/g)	White Rice	Red Rice	Black Rice	White Quinoa	Red Quinoa	Black Quinoa	White Teff	Brown Teff
Free ABTS	0.09 ± 0.02 ^a,A^	0.12 ± 0.01 ^b,B^	0.15 ± 0.01 ^c,C^	0.16 ± 0.03 ^c,A^	0.24 ± 0.04 ^d,B^	0.31 ± 0.03 ^e,C^	0.33 ± 0.02 ^f,A^	0.46 ± 0.02 ^g,B^
	21%	18%	19%	25%	23%	30%	31%	37%
Soluble conjugated ABTS	0.11 ± 0.01 ^a,A^	0.12 ± 0.02 ^a,A^	0.14 ± 0.01 ^b,B^	0.22 ± 0.03 ^c,A^	0.27 ± 0.02 ^d,e,B^	0.28 ± 0.01 ^d,B^	0.27 ± 0.01 ^d,e,A^	0.26 ± 0.01 ^e,A^
	26%	18%	18%	35%	26%	27%	25%	21%
Insoluble bound ABTS	0.22 ± 0.02 ^a,A^	0.42 ± 0.01 ^b,B^	0.47 ± 0.01 ^c,C^	0.25 ± 0.02 ^d,A^	0.53 ± 0.05 ^e,B^	0.44 ± 0.02 ^f,C^	0.48 ± 0.02 ^c,A^	0.52 ± 0.03 ^e,B^
	52%	64%	63%	40%	51%	43%	44%	42%
Total ABTS	0.42 ± 0.02 ^a,A^	0.66 ± 0.02 ^b,B^	0.77 ± 0.01 ^c,C^	0.63 ± 0.03 ^d,A^	1.04 ± 0.03 ^e,B^	1.03 ± 0.02 ^e,B^	1.08 ± 0.02 ^f,A^	1.24 ± 0.02 ^g,B^
Free DPPH	0.15 ± 0.02 ^a,A^	0.17 ± 0.01 ^b,B^	0.20 ± 0.01 ^c,C^	0.24 ± 0.03 ^d,A^	0.33 ± 0.02 ^e,B^	0.50 ± 0.02 ^f,C^	0.44 ± 0.02 ^g,A^	0.66 ± 0.02 ^h,B^
	21%	14%	14%	27%	22%	35%	47%	42%
Soluble conjugated DPPH	0.25 ± 0.03 ^a,A^	0.42 ± 0.01 ^b,B^	0.39 ± 0.03 ^c,C^	0.31 ± 0.02 ^d,A^	0.51 ± 0.12 ^e,B^	0.47 ± 0.11 ^f,C^	0.22 ± 0.03 ^g,A^	0.44 ± 0.03 ^h,B^
	35%	36%	28%	34%	33%	33%	24%	28%
Insoluble bound DPPH	0.32 ± 0.03 ^a,A^	0.59 ± 0.02 ^b,B^	0.79 ± 0.05 ^c,C^	0.35 ± 0.03 ^d,A^	0.69 ± 0.17 ^e,B^	0.45 ± 0.01 ^f,C^	0.27 ± 0.02 ^g,A^	0.49 ± 0.10 ^h,B^
	44%	50%	58%	39%	45%	32%	29%	30%
Total DPPH	0.72 ± 0.03 ^a,A^	1.18 ± 0.02 ^b,B^	1.38 ± 0.03 ^c,C^	0.90 ± 0.03 ^d,A^	1.53 ± 0.10 ^e,B^	1.42 ± 0.05 ^f,C^	0.93 ± 0.03 ^g,A^	1.59 ± 0.06 ^h,B^

Results are presented in dry weight as means ± SD, *n* = 5. Means within the line with at least one identical small superscript (in case of all types of flakes) and large superscript (in case of each type group of flakes) do not differ significantly (*p* ≥ 0.05), while means with various superscripts show a significant difference (*p* < 0.05). The percentage contributions of free, soluble bound and insoluble bound fractions to the antioxidant activity are presented in brackets. TE—Trolox equivalent; ABTS—2,2’-azinobis(3-ethylbenzo-thiazoline-6-sulfonic acid) diammonium salt; DPPH: 2,2-diphenyl-1-picrylhydrazyl.

**Table 4:** Results of individual phenolic concentrations in free phenolic fractions of non-traditional flakes.

Free Phenolics (µg/g)	White Rice	Red Rice	Black Rice	White Quinoa	Red Quinoa	Black Quinoa	White Teff	Brown Teff
Flavonoids								
Epigallocatechin	14.40 ± 0.10 ^a,A^	0.41 ± 0.02 ^b,B^	6.7 ± 0.20 ^c,C^	15.30 ± 0.10 ^d,A^	46.90 ± 0.20 ^e,B^	52.70 ± 1.00 ^f,C^	45.50 ± 0.20 ^g,A^	15.00 ± 0.20 ^h,B^
Catechin	2.02 ± 0.02 ^a,A^	15.20 ± 0.10 ^b,B^	2.05 ± 0.10 ^a,A^	32.10 ± 0.20 ^c,A^	33.30 ± 0.20 ^d,B^	18.70 ± 0.20 ^e,C^	5.49 ± 0.12 ^f,A^	2.83 ± 0.02 ^g,B^
Epicatechin	0.23 ± 0.02 ^a,A^	4.61 ± 0.05 ^b,B^	19.40 ± 0.20 ^c,C^	1.66 ± 0.03 ^d,A^	0.70 ± 0.03 ^e,B^	0.29 ± 0.01 ^f,C^	0.52 ± 0.04 ^g,A^	0.36 ± 0.03 ^h,B^
Rutin	24.10 ± 0.20 ^a,A^	28.0 ± 0.03 ^b,B^	15.8 ± 0.10 ^c,C^	57.50 ± 1.00 ^d,A^	129.0 ± 1.0 ^e,B^	84.80 ± 1.00 ^f,C^	24.70 ± 1.10 ^g,A^	43.50 ± 1.10 ^h,B^
Kaempferol	0.26 ± 0.02 ^a,A^	ND	0.68 ± 0.03 ^b,B^	2.77 ± 0.04 ^c,A^	1.18 ± 0.02 ^d, B^	0.48 ± 0.02 ^e,C^	0.16 ± 0.01 ^f,A^	1.70 ± 0.12 ^g,B^
Quercetin	ND	0.20 ± 0.02 ^a,A^	ND	2.55 ± 0.10 ^b,A^	1.41 ± 0.10 ^c,B^	1.80 ± 0.10 ^d,C^	ND	ND
Total free flavonoids	41.0 ± 0.2 ^a,A^	48.4 ± 0.1 ^b,B^	44.6 ± 0.1 ^c,C^	112.0 ± 1.0 ^d,A^	212.0 ± 1.0 ^e,B^	159.0 ± 1.0 ^f,C^	76.4 ± 1.0 ^g,A^	63.4 ± 1.0 ^h,B^
Phenolic acids								
Neochlorogenic acid	0.23 ± 0.03 ^a,A^	ND	ND	ND	0.08 ± 0.01 ^b,A^	1.71 ± 0.10 ^c,B^	1.37 ± 0.10 ^d,A^	0.50 ± 0.05 ^e,B^
Chlorogenic acid	1.54 ± 0.05 ^a,A^	1.25 ± 0.05 ^b,B^	7.66 ± 0.12 ^c,C^	1.74 ± 0.20 ^a,A^	2.58 ± 0.05 ^d,B^	3.61 ± 0.04 ^e,C^	10.00 ± 0.10 ^f,A^	2.14 ± 0.02 ^g,B^
Gallic acid	0.05 ± 0.01 ^a,A^	0.06 ± 0.01 ^a,A^	2.09 ± 0.02 ^b,B^	0.27 ± 0.04 ^c,A^	ND	ND	27.20 ± 1.0 ^d,A^	16.30 ± 0.10 ^e,B^
Protocatechuic acid	2.27 ± 0.05 ^a,A^	26.20 ± 0.5 ^b,B^	36.80 ± 0.20 ^c,C^	6.04 ± 0.10 ^d,A^	5.19 ± 0.06 ^e,B^	3.16 ± 0.04 ^f,C^	0.97 ± 0.02 ^g,A^	0.10 ± 0.01 ^h,B^
*p-*Hydroxybenzoic acid	0.78 ± 0.01 ^a,A^	0.30 ± 0.02 ^b,B^	0.15 ± 0.01 ^c,C^	9.23 ± 0.12 ^d,A^	ND	0.16 ± 0.01 ^c,B^	1.51 ± 0.03 ^e,A^	0.79 ± 0.03 ^a,B^
Vanillic acid	4.81 ± 0.10 ^a,A^	0.36 ± 0.04 ^b,B^	0.22 ± 0.03 ^c,C^	0.85 ± 0.02 ^d,A^	2.13 ± 0.10 ^e,B^	ND	ND	9.39 ± 0.20 ^f,A^
Caffeic acid	0.60 ± 0.03 ^a,A^	0.81 ± 0.01 ^b,B^	1.51 ± 0.10 ^c,C^	0.48 ± 0.03 ^d,A^	2.04 ± 0.01 ^e,B^	0.18 ± 0.01 ^f,C^	ND	ND
Syringic acid	0.04 ± 0.01 ^a,A^	0.04 ± 0.01 ^a,A^	ND	0.76 ± 0.02 ^b,A^	0.10 ± 0.01 ^c,B^	0.04 ± 0.01 ^a,C^	1.92 ± 0.08 ^d,A^	0.68 ± 0.03 ^e,B^
*p-*Coumaric acid	0.08 ± 0.01 ^a,A^	0.04 ± 0.01 ^b,B^	0.07 ± 0.02 ^a,A^	0.53 ± 0.02 ^c,A^	ND	ND	6.14 ± 0.10 ^d,A^	6.17 ± 0.02 ^d,A^
Ferulic acid	0.28 ± 0.01 ^a,A^	0.09 ± 0.01 ^b,B^	0.13 ± 0.01 ^c,C^	20.00 ± 0.10 ^d,A^	11.00 ± 0.10 ^e,B^	150.0 ± 3.0 ^f,C^	1.79 ± 0.05 ^g,A^	0.76 ± 0.02 ^h,B^
Sinapic acid	7.20 ± 0.05 ^a,A^	2.95 ± 0.10 ^b,B^	2.04 ± 0.20 ^c,C^	4.10 ± 2.00 ^d,A^	47.20 ± 1.10 ^e,B^	3.29 ± 0.10 ^d,C^	21.70 ± 0.9 ^f,A^	20.6 ± 0.03 ^f,A^
Ellagic acid	0.94 ± 0.02 ^a,A^	0.04 ± 0.01 ^b,B^	0.68 ± 0.02 ^c,C^	6.80 ± 1.30 ^d,A^	ND	0.73 ± 0.01 ^e,B^	9.61 ± 0.15 ^f,A^	9.45 ± 0.03 ^f,A^
*o-*Coumaric acid	0.38 ± 0.01 ^a,A^	ND	0.11 ± 0.01 ^b,B^	2.72 ± 0.02 ^c,A^	0.64 ± 0.02 ^d,B^	0.14 ± 0.01 ^e,C^	18.40 ± 0.20 ^f,A^	49.70 ± 2.20 ^g,B^
Protocatechin ethyl acid	10.00 ± 0.30 ^a,A^	1.47 ± 0.04 ^b,B^	1.99 ± 0.04 ^c,C^	0.83 ± 0.01 ^d,A^	1.34 ± 0.10 ^b,B^	1.16 ± 0.02 ^e,C^	51.20 ± 1.30 ^f,A^	3.42 ± 0.15 ^g,B^
Cinnamic acid	0.02 ± 0.01 ^a,A^	ND	ND	0.13 ± 0.01 ^b,A^	ND	0.15 ± 0.01 ^c,B^	0.62 ± 0.02 ^d,A^	ND
Total free phenolic acids	29.2 ± 0.2 ^a,A^	33.6 ± 0.5 ^b,B^	52.5 ± 0.2 ^c,C^	54.5 ± 1.0 ^d,A^	72.3 ± 1.0 ^e,B^	164.0 ± 3.0 ^f,C^	152.0 ± 1.0 ^f,A^	120.0 ± 2.0 ^g,B^
Total free phenolics	70.2 ± 0.2 ^a,A^	82.0 ± 0.4 ^b,B^	97.1 ± 0.2 ^c,C^	167.0 ± 1.0 ^d,A^	284.0 ± 1.0 ^e,B^	323.0 ± 2.0 ^f,C^	228.0 ± 1.0 ^g,A^	183.0 ± 2.0 ^h,B^

Results are presented in dry weight as means ± SD, *n* = 5. Means within the line with at least one identical small superscript (in case of all types of flakes) and large superscript (in case of each type group of flakes) do not differ significantly (*p* ≥ 0.05), while means with various superscripts show a significant difference (*p* < 0.05). ND—Not detected. Value of LOD: Quercetin and neochlorogenic, vanillic, caffeic, syringic, cinnamic acids 0.02 µg/g, kaempferol and gallic, *p-*hydroxybenzoic, *p-*coumaric, ellagic, *o-*coumaric acids 0.01 µg/g.

**Table 5:** Results of individual phenolic concentrations in soluble conjugated phenolic fractions of non-traditional flakes.

Soluble Conjugated Phenolics (µg/g)	White Rice	Red Rice	Black Rice	White Quinoa	Red Quinoa	Black Quinoa	White Teff	Brown Teff
Flavonoids								
Epigallocatechin	4.53 ± 0.10 ^a,A^	3.30 ± 0.10 ^b,B^	2.47 ± 0.10 ^c,C^	31.60 ± 1.20 ^d,A^	56.20 ± 1.00 ^e,B^	90.00 ± 1.0 ^f,C^	214.0 ± 2.0 ^g,A^	261.0 ± 3.0 ^h,B^
Catechin	33.50 ± 0.40 ^a,A^	17.40 ± 0.20 ^b,B^	16.70 ± 0.20 ^c,C^	10.50 ± 0.10 ^d,A^	47.90 ± 0.50 ^e,B^	33.10 ± 0.20 ^a,C^	32.2 ± 3.0 ^a,A^	13.2 ± 2.0 ^f,B^
Epicatechin	0.11 ± 0.01 ^a,A^	ND	ND	2.33 ± 0.03 ^b,A^	34.50 ± 0.30 ^c,B^	4.35 ± 0.25 ^d,C^	6.50 ± 0.05 ^e,A^	7.93 ± 0.20 ^f,B^
Rutin	1.58 ± 0.10 ^a,A^	0.68 ± 0.05 ^b,B^	3.27 ± 0.03 ^c,C^	9.64 ± 0.30 ^d,A^	1.99 ± 0.15 ^e,B^	6.95 ± 0.20 ^f,C^	81.00 ± 0.40 ^g,A^	8.90 ± 0.30 ^h,B^
Kaempferol	ND	ND	ND	0.46 ± 0.03 ^a,A^	1.10 ± 0.05 ^b,B^	0.56 ± 0.03 ^c,C^	ND	ND
Quercetin	ND	ND	ND	1.99 ± 0.10 ^a,A^	0.81 ± 0.03 ^b,B^	1.37 ± 0.06 ^c,C^	25.50 ± 0.30 ^d,^	ND
Total soluble conjugated flavonoids	39.8 ± 0.3 ^a,A^	21.4 ± 0.1 ^b,B^	22.4 ± 0.1 ^c,C^	56.5 ± 1.0 ^d,A^	143.0 ± 1.0 ^e,B^	136.0 ± 1.0 ^f,C^	359.0 ± 2.0 ^g,A^	291.0 ± 2.0 ^h,B^
Phenolic acids								
Neochlorogenic acid	13.30 ± 0.20 ^a,A^	1.60 ± 0.07 ^b,B^	0.37 ± 0.04 ^c,C^	1.78 ± 0.05 ^d,A^	3.77 ± 0.20 ^e,B^	3.62 ± 0.10 ^e,B^	13.30 ± 0.30 ^a,A^	20.00 ± 0.30 ^f,B^
Chlorogenic acid	15.10 ± 0.20 ^a,A^	4.45 ± 0.20 ^b,B^	14.80 ± 0.20 ^a,A^	11.2 ± 4.0 ^c,A^	0.07 ± 0.01 ^d,B^	15.10 ± 0.05 ^a,C^	2.55 ± 0.10 ^e,A^	2.51 ± 0.10 ^e,A^
Gallic acid	2.01 ± 0.02 ^a,A^	0.43 ± 0.03 ^b,B^	1.20 ± 0.05 ^c,C^	3.38 ± 0.10 ^d,A^	0.71 ± 0.05 ^e,B^	5.06 ± 0.10 ^f,C^	1.83 ± 0.05 ^g,A^	1.29 ± 0.10 ^c,B^
Protocatechuic acid	2.88 ± 0.10 ^a,A^	2.55 ± 0.05 ^b,B^	0.92 ± 0.05 ^c,C^	18.70 ± 0.10 ^d,A^	39.90 ± 0.5 ^e,B^	64.70 ± 0.30 ^f,C^	2.01 ± 0.05 ^g,A^	0.64 ± 0.02 ^h,B^
*p-*Hydroxybenzoic acid	7.48 ± 0.20 ^a,A^	2.09 ± 0.10 ^b,B^	4.69 ± 0.03 ^c,C^	16.00 ± 0.10 ^d,A^	0.47 ± 0.02 ^e,B^	1.31 ± 0.05 ^f,C^	0.44 ± 0.02 ^e,A^	0.29 ± 0.02 ^g,B^
Vanillic acid	3.10 ± 0.10 ^a,A^	0.99 ± 0.05 ^b,B^	1.61 ± 0.03 ^c,C^	2.12 ± 0.10 ^d,A^	40.40 ± 1.10 ^e,B^	55.30 ± 0.30 ^f, C^	5.28 ± 0.40 ^g A^	4.23 ± 0.10 ^h,B^
Caffeic acid	0.43 ± 0.02 ^a,A^	0.68 ± 0.10 ^b,B^	0.61 ± 0.10 ^b,B^	1.67 ± 0.10 ^c,A^	7.76 ± 0.15 ^d,B^	2.09 ± 0.05 ^e,C^	1.06 ± 0.02 ^f,A^	6.09 ± 0.08 ^g,B^
Syringic acid	7.07 ± 0.15 ^a,A^	2.15 ± 0.08 ^b,B^	2.22 ± 0.04 ^b,B^	4.12 ± 0.10 ^c,A^	3.72 ± 0.20 ^d,B^	0.80 ± 0.05 ^e C^	19.10 ± 0.20 ^f,A^	22.90 ± 0.20 ^g,B^
*p-*Coumaric acid	0.24 ± 0.02 ^a,A^	ND	ND	0.39 ± 0.03 ^b,A^	3.94 ± 0.20 ^c,B^	0.26 ± 0.03 ^a,C^	8.05 ± 0.15 ^d,A^	0.70 ± 0.05 ^e,B^
Ferulic acid	2.62 ± 0 ^a,A^	ND	ND	16.90 ± 0.30 ^b,A^	5.09 ± 0.10 ^c,B^	16.10 ± 0.20 ^d,C^	4.41 ± 0.15 ^e,A^	1.06 ± 0.10 ^f,B|^
Sinapic acid	ND	19.00 ± 0.20 ^a,A^	43.20 ± 0.30 ^b,B^	25.60 ± 1.0 ^c,A^	1.34 ± 0.04 ^d,B^	7.03 ± 0.15 ^e,C^	4.18 ± 0.10 ^f,A^	0.88 ± 0.05 ^g,B^
Ellagic acid	4.94 ± 0.10 ^a,A^	ND	0.49 ± 0.02 ^b,B^	30.90 ± 0.20 ^c,A^	0.64 ± 0.05 ^d,B^	0.79 ± 0.10 ^e,C^	ND	0.31 ± 0.02 ^f,A^
*o-*Coumaric acid	ND	ND	0.19 ± 0.02 ^a,A^	2.65 ± 0.10 ^b,A^	0.18 ± 0.02 ^a,B^	0.35 ± 0.05 ^c,C^	0.41 ± 0.05 ^c,A^	0.20 ± 0.02 ^a,B^
Protocatechin ethyl acid	8.51 ± 0.20 ^a,A^	ND	0.32 ± 0.02 ^b,B^	1.42 ± 0.05 ^c,A^	0.83 ± 0.08 ^d,B^	1.03 ± 0.02 ^e,C^	29.20 ± 0.20 ^f,A^	2.00 ± 0.05 ^g,B^
Cinnamic acid	ND	ND	ND	0.04 ± 0.01 ^a,A^	ND	0.43 ± 0.02 ^b,B^	ND	ND
Total soluble conjugated phenolic acids	67.7 ± 0.2 ^a,A^	33.9 ± 0.2 ^b,B^	70.6 ± 0.2 ^c,C^	137.0 ± 1.0 ^d,A^	109.0 ± 1.0 ^e,B^	174.0 ± 1.0 ^f,C^	91.8 ± 0.2 ^g,A^	63.1 ± 0.2 ^h,B^
Total soluble conjugated phenolics	108.0 ± 0.3 ^a,A^	55.3 ± 0.2 ^b,B^	93.0 ± 0.2 ^c,C^	194.0 ± 1.0 ^d,A^	252.0 ± 1.0 ^e,B^	310.0 ± 1.0 ^f,C^	451.0 ± 2.0 ^g,A^	354.0 ± 2.0 ^h,B^

Results are presented in dry weight as means ± SD, *n* = 5. Means within the line with at least one identical small superscript (in case of all types of flakes) and large superscript (in case of each type group of flakes) do not differ significantly (*p* ≥ 0.05), while means with various superscripts show a significant difference (*p* < 0.05). ND—Not detected. Value of LOD: Epicatechin, quercetin, protocatechin ethyl and cinnamic acids 0.02 µg/g, kaempferol and *p-*coumaric, ferulic, sinapic, ellagic, *o-*coumaric acids 0.01 µg/g.

**Table 6:** Results of individual phenolic concentrations in insoluble bound phenolic fractions of non-traditional flakes.

Insoluble Bound Phenolics (µg/g)	White Rice	Red Rice	Black Rice	White Quinoa	Red Quinoa	Black Quinoa	White Teff	Brown Teff
Flavonoids								
Epigallocatechin	2.99 ± 0.10 ^a,A^	4.92 ± 0.20 ^b,B^	3.00 ± 0.10 ^a,A^	5.68 ± 0.20 ^c,A^	35.90 ± 2.00 ^d,B^	40.20 ± 2.00 ^e,C^	13.4 ± 3.0 ^f,A^	45.50 ± 0.20 ^g,B^
Catechin	7.90 ± 0.15 ^a,A^	6.00 ± 0.20 ^b,B^	4.60 ± 0.20 ^c,C^	ND	13.60 ± 0.20 ^d,A^	10.60 ± 0.20 ^e,B^	62.30 ± 0.40 ^f,A^	31.80 ± 0.20 ^g,B^
Epicatechin	0.62 ± 0.05 ^a,A^	1.14 ± 0.08 ^b,B^	1.13 ± 0.05 ^b,B^	1.02 ± 0.05 ^c,A^	6.58 ± 0.12 ^d,B^	5.16 ± 0.14 ^e,C^	2.38 ± 0.20 ^f,A^	1.73 ± 0.15 ^g,B^
Rutin	2.35 ± 0.12 ^a,A^	0.60 ± 0.02 ^b,B^	2.00 ± 0.20 ^c,C^	ND	1.06 ± 0.05 ^d,A^	1.66 ± 0.04 ^e,B^	4.21 ± 0.12 ^f,A^	0.42 ± 0.04 ^g,B^
Kaempferol	ND	ND	ND	0.38 ± 0.03 ^a,A^	0.52 ± 0.02 ^b,B^	1.29 ± 0.10 ^c,C^	ND	ND
Quercetin	7.70 ± 1.00 ^a,A^	25.00 ± 0.40 ^b,B^	31.30 ± 2.00 ^c,C^	5.24 ± 0.05 ^d,A^	12.30 ± 1.50 ^e,B^	7.13 ± 0.12 ^a,C^	43.60 ± 1.30 ^f,A^	28.60 ± 1.00 ^g,B^
Total insoluble bound flavonoids	21.6 ± 3.0 ^a,A^	37.7 ± 0.3 ^b,B^	42.0 ± 1.0 ^c,C^	12.3 ± 0.1 ^d,A^	70.0 ± 2.0 ^e,B^	66.0 ± 1.0 ^f,C^	126.0 ± 3.0 ^g,A^	108.0 ± 1.0 ^h,B^
Phenolic acids								
Neochlorogenic acid	1.05 ± 0.03 ^a,A^	0.44 ± 0.05 ^b,B^	0.37 ± 0.02 ^c,C^	0.21 ± 0.04 ^d,A^	0.73 ± 0.05 ^e,B^	1.87 ± 0.10 ^f,C^	2.04 ± 0.12 ^f,A^	ND
Chlorogenic acid	1.92 ± 0.20 ^a,A^	3.08 ± 0.20 ^b,B^	11.50 ± 0.30 ^c C^	5.56 ± 0.15 ^d,A^	3.14 ± 0.06 ^b,B^	2.58 ± 0.05 ^e,C^	3.14 ± 0.13 ^b,A^	2.58 ± 0.12 ^e,B^
Gallic acid	0.79 ± 0.03 ^a,A^	0.54 ± 0.05 ^b,B^	0.75 ± 0.03 ^a,A^	0.23 ± 0.02 ^c,A^	1.17 ± 0.05 ^d,B^	1.50 ± 0.10 ^e,C^	3.04 ± 0.04 ^f,A^	0.73 ± 0.05 ^a,B^
Protocatechuic acid	0.34 ± 0.05 ^a,A^	0.99 ± 0.03 ^b,B^	1.59 ± 0.10 ^c,C^	0.93 ± 0.03 ^d,A^	5.67 ± 0.12 ^e,B^	5.21 ± 0.15 ^f,C^	1.76 ± 0.10 ^c,A^	ND
*p-*Hydroxybenzoic acid	0.64 ± 0.05 ^a,A^	0.77 ± 0.04 ^b,B^	1.07 ± 0.05 ^c,C^	1.09 ± 0.03 ^c,A^	0.25 ± 0.02 ^d,B^	ND	6.33 ± 0.10 ^e,A^	ND
Vanillic acid	2.48 ± 0.15 ^a,A^	2.35 ± 0.12 ^a,A^	0.99 ± 0.08 ^b,B^	2.06 ± 0.10 ^c,A^	5.11 ± 0.15 ^d,B^	0.19 ± 0.02 ^e,C^	3.89 ± 0.06 ^f,A^	1.34 ± 0.05 ^g,B^
Caffeic acid	0.09 ± 0.01 ^a,A^	0.22 ± 0.02 ^b,B^	0.55 ± 0.03 ^c,C^	0.09 ± 0.01 ^a,A^	0.97 ± 0.02 ^d,B^	0.27 ± 0.03 ^e,C^	ND	ND
Syringic acid	2.31 ± 0.20 ^a,A^	2.19 ± 0.10 ^a,A^	2.20 ± 0.08 ^a,A^	0.18 ± 0.01 ^b,A^	0.55 ± 0.02 ^c,B^	0.72 ± 0.03 ^d,C^	0.47 ± 0.02 ^e,A^	0.34 ± 0.02 ^f,B^
*p-*Coumaric acid	0.05 ± 0.01 ^a,A^	ND	ND	ND	ND	ND	0.45 ± 0.03 ^b,A^	0.22 ± 0.02 ^c,B^
Ferulic acid	20.60 ± 1.0 ^a,A^	228.0 ± 3.0 ^b,B^	257.0 ± 3.0 ^c,C^	1.26 ± 0.02 ^d,A^	12.40 ± 0.04 ^e,B^	26.50 ± 0.30 ^f,C^	93.3 ± 4.0 ^g,A^	111.0 ± 5.0 ^h,B^
Sinapic acid	ND	0.11 ± 0.01 ^a,A^	ND	1.42 ± 0.05 ^b,A^	3.76 ± 0.10 ^c,B^	0.21 ± 0.02 ^d,C^	26.2 ± 0.20 ^e,A^	47.5 ± 0.40 ^f,B^
Ellagic acid	1.09 ± 0.03 ^a,A^	15.40 ± 0.20 ^b,B^	ND	0.49 ± 0.05 ^c,A^	0.32 ± 0.03 ^d,B^	ND	1.24 ± 0.02 ^e,A^	ND
*o-*Coumaric acid	0.24 ± 0.02 ^a,A^	0.06 ± 0.01 ^b,B^	ND	0.09 ± 0.01 ^c,A^	1.38 ± 0.03 ^d,B^	0.08 ± 0.01 ^c,A^	0.17 ± 0.02 ^e,A^	0.03 ± 0.01 ^f,B^
Protocatechin ethyl acid	2.79 ± 0.10 ^a,A^	0.26 ± 0.02 ^b,B^	1.25 ± 0.05 ^c,C^	ND	0.14 ± 0.02 ^d,A^	0.15 ± 0.02 ^d,A^	3.01 ± 0.10 ^e,A^	1.18 ± 0.03 ^c,B^
Cinnamic acid	ND	ND	ND	ND	ND	0.07 ± 0.01 ^a,A^	ND	0.08 ± 0.01 ^a,A^
Total insoluble bound phenolic acids	34.4 ± 1.0 ^a,A^	254.0 ± 2.0 ^b,B^	277.0 ± 3.0 ^c,C^	25.9 ± 0.1 ^d,A^	35.6 ± 0.1 ^a,B^	39.4 ± 0.2 ^e,C^	145.0 ± 4.0 ^f,A^	165.0 ± 5.0 ^g,B^
Total insoluble bound phenolics	56.0 ± 2.0 ^a,A^	292.0 ± 2.0 ^b,B^	319.0 ± 2.0 ^c,C^	38.2 ± 0.1 ^d,A^	106.0 ± 2.0 ^e,B^	105.0 ± 1.0 ^e,C^	271.0 ± 3.0 ^f,A^	273.0 ± 4.0 ^g,B^

Results are presented in dry weight as means ± SD, *n* = 5. Means within the line with at least one identical small superscript (in case of all types of flakes) and large superscript (in case of each type group of flakes) do not differ significantly (*p* ≥ 0.05), while means with various superscripts show a significant difference (*p* < 0.05). ND—Not detected. Value of LOD: Catechin, rutin, protocatechin ethyl, neochlorogenic, protocatechuic, caffeic, cinnamic acids 0.02 µg/g, kaempferol and *p-*hydroxybenzoic, *p-*coumaric, sinapic, ellagic, *o-*coumaric acids 0.01 µg/g.

## Supplementary Materials

The following are available online at https://www.mdpi.com/2076-3921/8/11/565/s1. Figure S1: High performance liquid chromatography (HPLC) chromatogram for analysis of sugars in teff from Bolivia. Figure S2: HPLC chromatogram for analysis of free phenolics in black quinoa from Peru; detected at the wavelength of 275 nm. Figure S3: HPLC chromatogram for analysis of soluble conjugated phenolics in black quinoa from Peru; detected at the wavelength of 275 nm. Figure S4: HPLC chromatogram for analysis of insoluble bound phenolics in black quinoa from Peru; detected at the wavelength of 275 nm. Table S1: The relation of several evaluation parameters. Table S2: Results of total individual phenolic concentrations in phenolic fractions of non-traditional flakes. Table S3: The relation of several evaluation parameters for individual phenolics.
