# Supplementary material for: The Nutritional Value of Non-Traditional Gluten-Free Flakes and Their Antioxidant Activity
Source: Antioxidants (Basel). 2019 Nov 16;8(11):565. doi: 10.3390/antiox8110565 (PMC6912764; doi:10.3390/antiox8110565)
Supplement: Supplementary file 1 [file antioxidants-08-00565-s001.pdf]

antioxidants

Supplementary data

The Nutritional Value of Non-Traditional Gluten-Free Flakes and Their Antioxidant Activity

Kristýna Šťastná [1], Martina Mrázková [1], Daniela Sumczynski [1,*], Betül Cındık [2] and Erkan Yalçın [2]

[1] Department of Food Analysis and Chemistry, Tomas Bata University in Zlín, Vavrečkova 275, Zlín 760 01, Czech Republic; kstastna@utb.cz (K.S.); mmrazkova@utb.cz (M.M.)
[2] Department of Food Engineering, Bolu Abant Ízzet Baysal University, Gölköy Campus, Bolu 14030, Turkey; betul1506@hotmail.com (B.C.); erkanyalcin93@hotmail.com (E.Y.)
* Correspondence: sumczynski@utb.cz

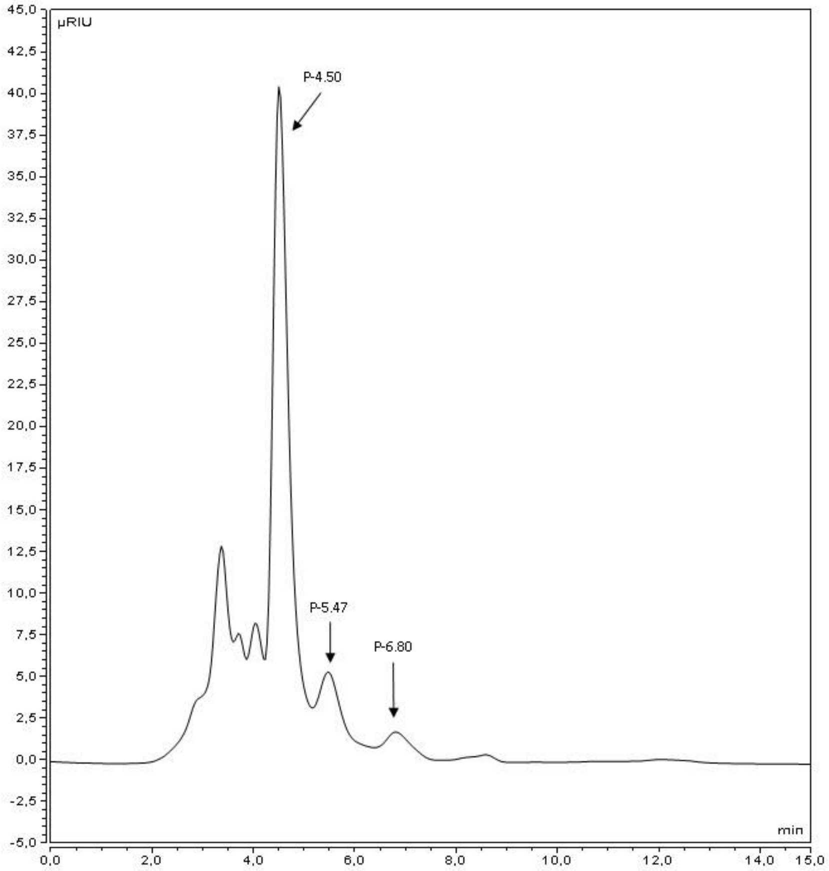

Figure S1. High performance liquid chromatography (HPLC) chromatogram for analysis of sugars in teff from Bolivia.

P-4.50 (D(+)-maltose), P-5.47 (D(+)-glucose), P-6.80 (D(-)-fructose).

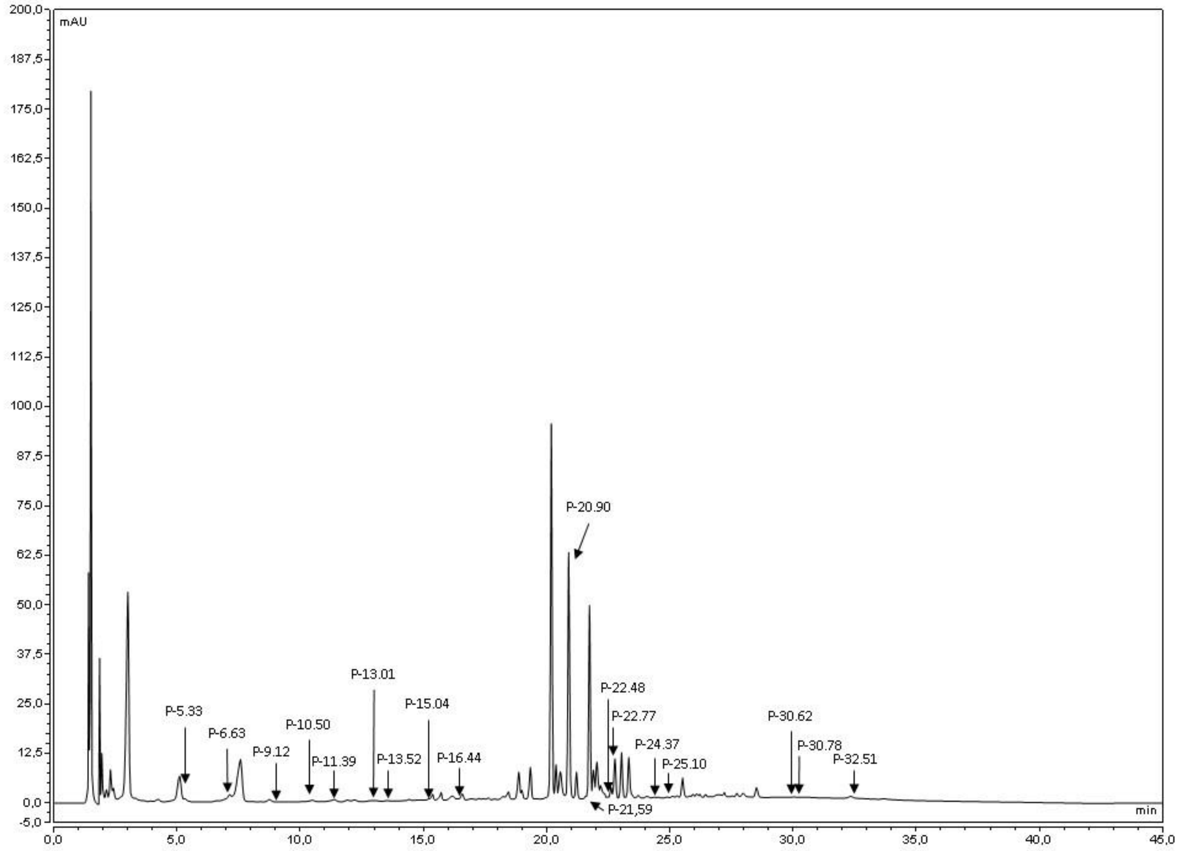

Figure S2. HPLC chromatogram for analysis of free phenolics in black quinoa from Peru; detected at the wavelength of 275 nm.

P-5.33 (protocatechuic acid), P-6.63 (neochlorogenic acid), P-9.12 (*p*-hydroxybenzoic acid), P-10.50 (epigalocatechin), P-11.39 (catechin), P-13.01 (chlorogenic acid), P-13.52 (caffeic acid), P-15.04 (syringic acid), P-16.44 (epicatechin), P-20.90 (ferulic acid), P-21.59 (sinapic acid), P-22.48 (ellagic acid), P-22.77 (rutin), P-24.37 (*o*-coumaric acid), P-25.10 (protocatechin ethyl acid), P-30.62 (cinnamic acid), P-30.78 (kaempferol), P-32.51 (quercetin).

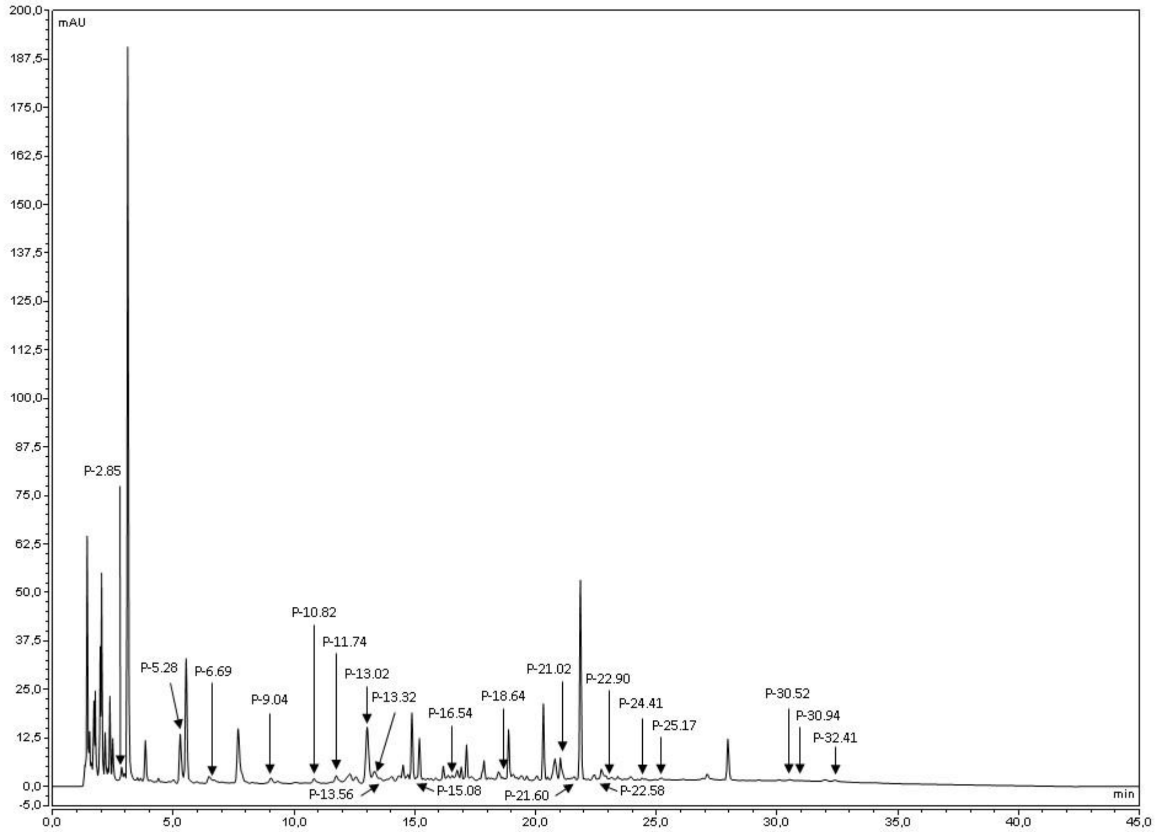

Figure S3. HPLC chromatogram for analysis of soluble conjugated phenolics in black quinoa from Peru; detected at the wavelength of 275 nm.

P-2.85 (gallic acid), P-5.28 (protocatechuic acid), P-6.69 (neochlorogenic acid), P-9.04 (*p*-hydroxybenzoic acid), P-10.82 (epigalocatechin), P-11.74 (catechin), P-13.02 (vanilic acid), P-13.32 (chlorogenic acid), P-13.56 (caffeic acid), P-15.08 (syringic acid), P-16.54 (epicatechin), P-18.64 (*p*-coumaric acid), P-21.02 (ferulic acid), P-21.60 (sinapic acid), P-22.58 (ellagic acid), P-22.90 (rutin), P-24.41 (*o*-coumaric acid), P-25.17 (protocatechin ethyl acid), P-30.52 (cinnamic acid), P-30.94 (kaempferol), P-32.41 (quercetin).

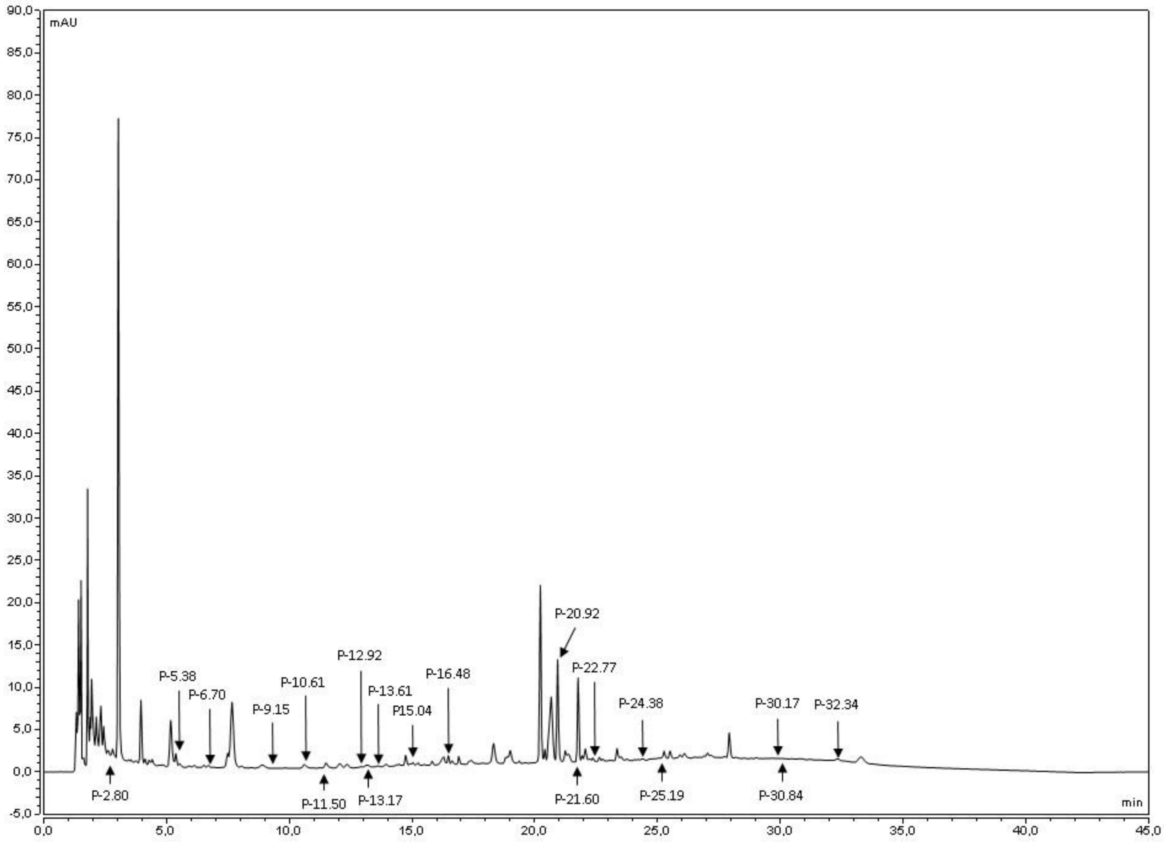

Figure S4. HPLC chromatogram for analysis of insoluble bound phenolics in black quinoa from Peru; detected at the wavelength of 275 nm.

P-2.80 (gallic acid), P-5.38 (protocatechuic acid), P-6.70 (neochlorogenic acid), P-10.61 (epigalocatechin), P-11.50 (catechin), P-12,92 (vanilic acid), P-13.17 (chlorogenic acid), P-13.61 (caffeic acid), P-15.04 (syringic acid), P-16.48 (epicatechin), P-20.92 (ferulic acid), P-21.60 (sinapic acid), P-22.77 (rutin), P-24.38 (*o*-coumaric acid), P-25.19 (protocatechin ethyl acid), P-30.17 (cinnamic acid), P-30.84 (kaempferol), P-32.34 (quercetin).

Table S1. The relation of several evaluation parameters

**Parameter *r***	**Ash**	**Lipids**	**Crude Protein**	**Starch**	**Resistant Starch**	**CF**	**NDF**	**TDF**
DMD	−0.6172	−0.3910	−0.7117	0.7755	0.2911	0.3349	−0.7277	−0.9531
OMD	−0.5712	−0.4060	−0.6814	0.7604	0.3503	0.4786	−0.6004	−0.9419
	**Free TFC**	**Soluble Bound TFC**	**Insoluble Bound TFC**	**Total TFC**	**Free TPC**	**Soluble Bound TPC**	**Insoluble Bound TPC**	**Total TPC**
Free ABTS	0.4827	*	*	*	0.9262	*	*	*
Soluble conjugated ABTS	*	0.8353	*	*	*	0.9861	*	*
Insoluble bound ABTS	*	*	0.6947	*	*	*	0.6487	*
Total ABTS	*	*	*	0.9247	*	*	*	0.9371
Free DPPH	0.4726				0.9023	*	*	*
Soluble conjugated DPPH	*	−0.0695	*	*	*	0.3795	*	*
Insoluble bound DPPH	*	*	0.0664	*	*	*	0.6350	*
Total DPPH	*	*	*	0.5914	*	*	*	0.7604

r—Pearson´s correlations coefficient, CF—Crude fibre, NDF—Neutral-detergent fibre, TDF—Total dietary fibre, DMD—Dry matter digestibility, OMD—Organic matter digestibility, TFC—Total flavonoid content, TPC—Total phenolic content; ABTS—2,2′-azinobis(3-ethylbenzo-thiazoline-6-sulfonic acid) diammonium salt, DPPH—radical 2,2-diphenyl-1-picrylhydrazyl.

Table S2. Results of total individual phenolic concentrations in phenolic fractions of non-traditional flakes.

**Total Individual Phenolics (μg/g)**	**White Rice**	**Red Rice**	**Black Rice**	**White Quinoa**	**Red Quinoa**	**Black Quinoa**	**White Teff**	**Brown Teff**
Flavonoids								
Epigallocatechin	21.9 ± 0.2 ^{a,A}	8.63 ± 0.02 ^{b,B}	12.2 ± 0.2 ^{c,C}	52.6 ± 1.0 ^{d,A}	139.0 ± 4.0 ^{e,B}	182.0 ± 5.0 ^{f,C}	272.0 ± 5.0 ^{g,A}	322.0 ± 6.0 ^{h,B}
Catechin	43.4 ± 0.3 ^{a,A}	38.6 ± 0.4 ^{b,B}	23.4 ± 0.2 ^{c,C}	42.6 ± 0.4 ^{d,A}	94.8 ± 1.0 ^{e,B}	62.4 ± 0.5 ^{f,C}	100.0 ± 2.0 ^{g,A}	47.8 ± 1.0 ^{h,B}
Epicatechin	0.96 ± 0.04 ^{a,A}	5.75 ± 0.10 ^{b,B}	20.4 ± 0.5 ^{c,C}	5.01 ± 0.03 ^{d,A}	41.8 ± 0.5 ^{e,B}	9.80 ± 0.02 ^{f,C}	9.40 ± 0.10 ^{g,A}	10.0 ± 0.20 ^{f,B}
Rutin	28.0 ± 0.5 ^{a,A}	29.3 ± 0.2 ^{b,B}	21.1 ± 0.3 ^{c,C}	67.1 ± 1.1 ^{d,A}	132.0 ± 3.0 ^{e,B}	93.4 ± 1.2 ^{f,C}	109.9 ± 2.2 ^{g,A}	52.8 ± 1.0 ^{h,B}
Kaempferol	0.26 ± 0.03 ^{a,A}	ND	0.68 ± 0.05 ^{b,B}	3.61 ± 0.10 ^{c,A}	2.80 ± 0.05 ^{d,B}	2.33 ± 0.04 ^{e,C}	0.16 ± 0.02 ^{f,A}	1.70 ± 0.05 ^{g,B}
Quercetin	7.70 ± 0.10 ^{a,A}	25.2 ± 0.4 ^{b,B}	31.3 ± 0.8 ^{c,C}	9.78 ± 0.05 ^{d,A}	14.5 ± 0.8 ^{e,B}	10.3 ± 0.4 ^{f,C}	69.1 ± 0.9 ^{g,A}	28.6 ± 0.4 ^{h,B}
Total individual flavonoids	102.0 ± 0.4 ^{a,A}	108.0 ± 0.3 ^{b,B}	109.0 ± 0.6 ^{c,C}	181.0 ± 1.0 ^{d,A}	425.0 ± 3.0 ^{e,B}	360.0 ± 4.0 ^{f,C}	561.0 ± 4.0 ^{g,A}	463.0 ± 5.0 ^{h,B}
Phenolic acids								
Neochlorogenic acid	14.60 ± 0.20^{a,A}	2.04 ± 0.02^{b,B}	0.74 ± 0.03^{c,C}	1.99 ± 0.05^{b,A}	4.58 ± 0.06^{d,B}	7.20 ± 0.10^{e,C}	16.70 ± 0.20^{f,A}	20.50 ± 0.30^{g,B}
Chlorogenic acid	18.60 ± 0.30^{a,A}	8.78 ± 0.15^{b,B}	34.00 ± 0.50^{c,C}	18.50 ± 0.30^{a,A}	5.79 ± 0.04^{d,B}	21.30 ± 0.50^{e,C}	15.70 ± 1.00^{f,A}	7.23 ± 0.08^{g,B}
Gallic acid	2.85 ± 0.05^{a,A}	1.03 ± 0.03^{b,B}	4.04 ± 0.02^{c,C}	3.88 ± 0.05^{d,A}	1.88 ± 0.04^{e,B}	6.56 ± 0.10^{f,C}	32.10 ± 0.30^{g,A}	18.30 ± 0.50^{h,B}
Protocatechuic acid	5.49 ± 0.04^{a,A}	29.70 ± 1.00^{b,B}	39.30 ± 1.0^{c,C}	25.7 ± 0.7^{d,A}	50.8 ± 1.5^{e,B}	73.10 ± 1.50^{f,C}	4.74 ± 0.05^{g,A}	0.74 ± 0.02^{h,B}
p-Hydroxybenzoic acid	8.90 ± 0.50^{a,A}	3.16 ± 0.04^{b,B}	5.91 ± 0.04^{c,C}	26.30 ± 0.20^{d,A}	0.72 ± 0.05^{e,B}	1.47 ± 0.05^{f,C}	8.28 ± 0.10^{a,A}	1.08 ± 0.04^{g,B}
Vanillic acid	10.40 ± 0.20^{a,A}	3.70 ± 0.05^{b,B}	2.82 ± 0.05^{c,C}	5.03 ± 0.04^{d,A}	47.6 ± 1.0^{e,B}	55.5 ± 1.5^{f,C}	9.17 ± 0.20^{g,A}	15.00 ± 0.30^{h,B}
Caffeic acid	1.12 ± 0.02^{a,A}	1.71 ± 0.04^{b,B}	2.67 ± 0.05^{c,C}	2.24 ± 0.04^{d,A}	10.8 ± 0.1^{e,B}	2.54 ± 0.05^{f,C}	1.06 ± 0.04^{g,A}	6.09 ± 0.04^{h,B}
Syringic acid	9.42 ± 0.20^{a,A}	4.38 ± 0.10^{b,B}	4.42 ± 0.10^{b,B}	5.06 ± 0.05^{c,A}	4.37 ± 0.05^{b,B}	1.56 ± 0.10^{d,C}	21.50 ± 0.30^{e,A}	23.90 ± 0.30^{f,B}
p-Coumaric acid	0.37 ± 0.02^{a,A}	0.04 ± 0.01^{b,B}	0.07 ± 0.01^{c,C}	0.92 ± 0.02^{d,A}	3.94 ± 0.15^{e,B}	0.26 ± 0.04^{f,C}	87.10 ± 1.40^{g,A}	7.09 ± 0.07^{h,B}
Ferulic acid	23.50 ± 0.80^{a,A}	228.0 ± 5.0^{b,B}	257.0 ± 5.0^{c,C}	38.20 ± 0.70^{d,A}	28.5 ± 0.8^{e,B}	193.0 ± 5.0^{f,C}	6.20 ± 0.30^{g,A}	1.82 ± 0.04^{h,B}
Sinapic acid	7.20 ± 0.30^{a,A}	22.10 ± 0.40^{b,B}	45.20 ± 0.50^{c,C}	31.10 ± 1.0^{d,A}	52.3 ± 1.1^{e,B}	10.50 ± 0.07^{f,C}	52.10 ± 1.10^{e,A}	79.00 ± 2.00^{g,B}
Ellagic acid	6.97 ± 0.05^{a,A}	15.40 ± 0.50^{b,B}	1.17 ± 0.10^{c,C}	38.20 ± 0.60^{d,A}	0.96 ± 0.05^{e,B}	1.52 ± 0.07^{f,C}	10.9 ± 0.2^{g,A}	9.76 ± 0.12^{h,B}
o-Coumaric acid	0.62 ± 0.04^{a,A}	0.06 ± 0.01^{b,B}	0.30 ± 0.02^{c,C}	5.46 ± 0.10^{d,A}	2.20 ± 0.10^{e,B}	0.57 ± 0.04^{a,C}	19.00 ± 0.20^{f,A}	49.90 ± 0.50^{g,B}
Protocatechin ethyl acid	21.30 ± 0.50^{a,A}	1.73 ± 0.05^{b,B}	3.56 ± 0.30^{c,C}	2.25 ± 0.02^{d,A}	2.31 ± 0.02^{e,B}	2.34 ± 0.04^{e,B}	83.40 ± 1.00^{f,A}	6.60 ± 0.10^{g,B}
Cinnamic acid	0.02 ± 0.01^{a,A}	ND	ND	0.17 ± 0.02^{b,A}	ND	0.65 ± 0.05^{c,B}	0.70 ± 0.05^{c,A}	ND

Total individual phenolic acids	131.0 ± 0.7^{a,A}	322.0 ± 4.0^{b,B}	400.0 ± 4.0^{c,C}	216.0 ± 1.0^{d,A}	217.0 ± 1.0^{d,A}	377.0 ± 4.0^{e,B}	296.0 ± 1.0^{f,A}	237.0 ± 2.0^{g,B}
Total individual phenolics	233.0 ± 1.0^{a,A}	430.0 ± 4.0^{b,B}	509.0 ± 4.0^{c,C}	397.0 ± 1.0^{d,A}	642.0 ± 3.0^{e,B}	737.0 ± 4.0^{f,C}	857.0 ± 4.0^{g,A}	700.0 ± 4.0$^{h\ B}$

Results are presented in dry weight as means ± SD, $n = 5$. Means within the line with at least one identical small superscript (in case of all types of flakes) and large superscript (in case of each type group of flakes) do not differ significantly ($p \geq 0.05$), while means with various superscripts show a significant difference ($p < 0.05$). ND—Not detected. Value of LOD: cinnamic acid 0.02 μg/g, kaempferol 0.01 μg/g.

Table S3. The relation of several evaluation parameters for individual phenolics.

**Parameter *r***	**EG**	**CA**	**EC**	**RU**	**KA**	**QU**	**Total Flavonoids**	**NeA**	**ChA**
Free ABTS	0.4713	–0.1388	–0.3608	0.2266	0.0532	*	0.2304	*	0.2237
Soluble conjugated ABTS	0.6906	0.3077	0.4342	0.4083	*	*	0.7373	0.2323	–0.4256
Insoluble bound ABTS	0.6114	0.3447	0.5330	–0.2110	*	0.5792	0.7479	0.2620	0.0828
Total ABTS	0.8942	0.5583	0.4146	0.6084	0.2225	0.4319	0.8912	0.4215	–0.3526
Free DPPH	0.4910	–0.1153	–0.4035	0.2608	0.0571	0.3899	0.2600	0.4188	0.1450
Soluble conjugated DPPH	0.5448	0.1444	0.6291	–0.5956	*	*	–0.1394	–0.3137	–0.2234
Insoluble bound DPPH	0.3905	–0.5924	0.2414	–0.5574	*	0.1008	–0.1383	–0.5229	0.5819
Total DPPH	0.6838	0.0587	0.6210	0.2268	0.2896	–0.0693	0.3526	–0.0779	–0.1984
	**GA**	**PA**	***p*-HA**	**VA**	**CaA**	**SA**	***p*-CA**	**FA**	**SiA**
Free ABTS	0.8186	–0.5238	–0.1752	0.7684	–0.0081	0.4319	0.9493	0.2295	0.4010
Soluble conjugated ABTS	0.3810	0.5604	–0.3237	0.5935	0.6100	0.3768	0.2808	0.2228	–0.7816
Insoluble bound ABTS	0.3979	0.6430	0.2710	0.1570	0.8253	–0.2743	*	0.3623	0.4810
Total ABTS	0.6039	0.1060	–0.5090	0.4734	0.5159	0.5018	0.3783	–0.2275	0.7158
Free DPPH	0.7795	–0.5599	–0.1741	0.8026	–0.1761	0.3421	0.9328	0.3466	0.3322
Soluble conjugated DPPH	0.3677	0.5506	–0.4282	0.6113	0.6458	–0.3205	–0.5647	0.1315	–0.1974
Insoluble bound DPPH	–0.3674	0.3310	–0.5315	–0.0432	0.7766	0.3354	*	0.5751	–0.2092
Total DPPH	–0.0696	0.4629	–0.6664	0.5148	0.6969	–0.0250	–0.2981	0.2632	0.5323

	**EA**	***o*-CA**	**PeA**	**CiA**	**Total phenolic acids**	**Total Phenolics**
Free ABTS	0.6896	0.8209	0.2637	*	0.8288	0.6178
Soluble conjugated ABTS	–0.0541	–0.1393	0.1419	*	0.6433	0.8854
Insoluble bound ABTS	*	0.3480	–0.4628	*	0.2158	0.5118
Total ABTS	–0.3438	0.6509	0.2055	0.3698	0.2909	0.9261

Free DPPH	0.6210	0.7816	0.1872	0.5782	0.8475	0.6482
Soluble conjugated DPPH	–0.5307	–0.4196	–0.7232	*	0.1622	–0.0770
Insoluble bound DPPH	*	–0.6054	–0.6704	*	0.6665	0.6238
Total DPPH	–0.4974	0.3245	–0.4643	–0.1687	0.4423	0.5092

EG—epigallocatechin, CA—catechin, EC—epicatechin, RU—rutin, KA—kaempferol, QU—quercetin, NeA—neochlorogenic acid, ChA—chlorogenic acid, GA—gallic acid, PA—protocatechuic acid, *p*-HA—*p*-hydroxybenzoic acid, VA—vanillic acid, CaA—caffeic acid, SA—syringic acid, *p*-CA—*p*-coumaric acid, FA—ferulic acid, SiA—sinapic acid, EA—ellagic acid, *o*-CA—*o*-coumaric acid, PeA—protocatechin ethyl acid, CiA—cinnamic acid
